# Impact of Fly Ashes from Combustion in Fluidized Bed Boilers and Siliceous Fly Ashes on Durability of Mortars Exposed to Seawater and Carbonation Process

**DOI:** 10.3390/ma14092345

**Published:** 2021-04-30

**Authors:** Elżbieta Janowska-Renkas, Agnieszka Kaliciak

**Affiliations:** Faculty of Civil Engineering and Architecture, Opole University of Technology, 45-758 Opole, Poland; ag.kaliciak@doktorant.po.edu.pl

**Keywords:** siliceous fly ash, FBC fly ash, mortars, seawater corrosion, carbonation process

## Abstract

This article presents test results of aggressive environment impact, i.e., seawater, acid solutions and carbonation, on the durability of cement–ash mortars. Tests were conducted on CEM I 42.5R-based mortars containing 35 to 70% by mass of FBC fly ash from brown and black coal combustion in a homogeneous form and mixtures of 35% by mass of siliceous fly ashes (CFA) and 35% by mass of FBC fly ash. It was demonstrated that in normal conditions (20 °C), FBC ashes showed higher pozzolanic activity than CFA, except when their curing temperature was increased to 50 °C. FBC ashes increased mortars’ water demands, which led to an accelerated carbonation process. In an environment of Cl^-^ ions, cement–ash mortars showed more Ca^2+^ ions leached and no expansive linear and mass changes, which, with their increased strength, might be an argument in favour for their future use in construction of coastal structures resistant to seawater. FBC ash content may be increased to 35% by mass, maintaining mortars’ resistance to seawater, acid rain and carbonation. A favourable solution turned out to be a FBC and CFA mixed addition to cement of 35% by mass each, in contrast to mortars containing 70% of FBC fly ash in homogeneous form.

## 1. Introduction

Fluidized bed combustion (FBC) fly ashes are byproducts of the combustion process in power plants and combined heat and power plants in Poland and the world, where burning at a lower temperature (ca. 850 °C) is applied [1,2]. Materials such as lignite and hard coal, as well as biomass, are burned [1]. Depending on the quality and type of fuel, as well as the sorbents used in a furnace in the form of calcium carbonate or dolomite, FBC fly ashes have different properties [2].

The morphology of FBC fly ash grains is different from that of fly ashes obtained in pulverized fuel-fired boilers. Many papers, including [1,3,4,5], demonstrate that FBC fly ashes contain more hydrated aluminosilicate substance, anhydrite, calcium oxide and quartz compared to fly ashes coming from combustion in pulverized fuel-fired boilers. Particles of siliceous fly ash have a spherical shape with a smooth surface and a glassy matrix, in contrast to FBC fly ash particles, which have an irregular shape and surface, and which often occur in compacted forms [1,3,6,7,8].

Due to high loss on ignition (LOI) values and high sulphate content, FBC fly ashes currently do not meet requirements of PN-EN 450-1 standard ”Fly ash for concrete”, which prevents their use to cement [1]. During contact of FBC fly ashes with water, the ashes harden and a high quantity of heat is generated, and the pH value of the solution changes to alkaline (pH ca. 12–13), which leads to hydration of the anhydrite. According to [8,9], a consequence of the solution’s pH value change is the crystallization of calcium hydroxide with sulphate ions and aluminium. As a result of this reaction, the formation of expansive ettringite takes place, which leads to deformation and cracks in the microstructure of the paste containing FBC fly ash, when other cement ingredients have already formed hydration products shaping the hardened structure [10]. The tendency of cement pastes containing FBC fly ashes to expand was also confirmed in papers by Chen et al. [1,10].

When sulphate ions are used up, ettringite may convert to a monosulphate [1,8,11,12]. Refs. [13,14] observed that the application of cement with a higher SO_3_ content for production of concrete elements of coastal structures may delay expansion in the concrete.

This results from the fact that the reaction between sulphate ions and C_3_A and C-S-H phases occurs in water. A product of these reactions is ettringite that does not cause expansion, because in the presence of chlorides, calcium ions, which constitute a significant part of ettringite, are washed away from cement-based materials with seawater.

Therefore, FBC fly ashes, due to their high content of sulphur oxides, may become an essential mineral additive to the cement, and their presence may contribute to delayed destruction of concrete elements of coastal structures due to the increased content of ettringite, which gets crystallized in materials containing FBC fly ashes [13]. It was demonstrated that chlorides originating from seawater reacted with ettringite, causing formation of a chloride equivalent of the ettringite (C_3_A∙3CaCl_2_∙30H_2_O), which might be washed out with seawater from cementitious materials with addition of fly ashes.

Refs. [13,15] demonstrated weakened microstructure in cementitious materials revealed by carbonation under atmospheric CO_2_. According to Neville [13], the rate of the carbonation process in materials based on cement binders containing fly ashes depends on the material microstructure and properties of the hardened paste. In the case of fly-ash-containing binders with high silica content, their high reactivity with calcium hydroxide Ca(OH)_2_ was observed along with the formation of an additional product in the form of the C-S-H phase. Therefore, cement–ash materials showed lower crystalline calcium hydroxide (portlandite) content, and thus increased resistance to the impact of aggressive environment.

According to Bier [16], a small amount of portlandite formed in the cement hydration process reacts with a small amount of CO_2_, which has a direct impact on limiting destructive changes in cementitious materials occurring as a result of the carbonation process. Furthermore, this author [16] claims that the lower the portlandite content in the cementitious material is, the faster the carbonation process occurs. 

Patel et al. [17] claim that the presence of siliceous fly ash in cement binders causes the formation of a tighter microstructure. In their opinion, it has an influence on limiting the diffusion of aggressive ions in cement–ash materials and lowering rate of their carbonation. Therefore, the carbonation rate of cement–ash materials depends on many factors, including the chemical composition of the binder, the quantity and type of fly ashes used, the silicate content and the microstructure of the hardened paste. FBC fly ashes show a partial sequestration, i.e., they capture a carbon dioxide from air, which is confirmed by [17,18,19]. 

To improve the durability and assure the safety of concrete structures, several factors are taken into account, starting from: the origin of raw materials; the production of materials and components up to workmanship, use, maintenance and repairs; demolition works and recycling or reuse of elements. Requirements that limit the environmental impact of structures should be already declared at the stage of planning and designing [20,21]. However, present guidelines and requirements for designing concrete structures pay little attention to environmental aspects [22].

Furthermore, possible management of industrial waste (such as fly ash, blast furnace slag, silica fume or metakaolin), including the recycling of more fly ash from fluidized bed or pulverized coal-fired boilers (i.e., a difficult industrial waste generated in power plants and combined heat and power plants), additionally meets the requirements of EU directives on sustainable development policy. Reduction of clinker consumption and its replacement with FBC fly ash in cement composition has a direct impact on reducing the effects of global warming, the consumption of energy and natural resources and the emission of waste or air pollution. At present, the application of FBC fly ashes to cement is still problematic, as there are no settled standards on their possible use in production of mortars or concretes due to the instability of their composition or their higher SO_3_ content [1,2,3,4,8,12,23].

Therefore, it seemed reasonable to focus research on determination of the impact of aggressive environment, including corrosion that occurs under the influence of seawater and the carbonation process of concretes and mortars containing FBC fly ashes. The aim of the study was to determine the durability of mortars containing 35 to 70% by mass of FBC fly ashes, originating from three different power plants; with different content of SiO_2_ (35.87 to 43.78%) and SO_3_ (3.09 to 4.68%); used in a homogeneous form, i.e., “pure” ash, and blends of FBC and siliceous fly ashes; in the seawater environment and in accelerated carbonation. It should be emphasized that so far, the beneficial impact of lower FBC fly ash content (30%) on improved performance parameters of mortars containing that ash has been demonstrated [6,8,24].

## 2. Material for Testing

Three types of FBC fly ashes were used for testing: FBC I—originating from lignite combustion in a CHP Plant, FBC II and FBC III—obtained from hard coal combustion in two power plants. Siliceous, high-calcium fly ashes (CFA) were obtained from a power plant from hard coal combustion in pulverized coal-fired boilers. Mortars were prepared on the basis of Portland cement CEM I 42.5R without and with the addition of different quantities of FBC II and FBC III fly ashes, as well as mixtures of FBC and siliceous (CFA) fly ashes. Fine aggregate (sand) with particle size 0–2 mm was applied to mortars. The composition and ratios of these mortars are given in Table 1.

The composition of the mortars was determined in such a way that the largest quantity of FBC fly ash could be utilized; its content in the cement ranged from 35 to 70% by mass.

The resistance of mortars to corrosion was determined after 28 days of their curing in tap water. The mortars were exposed to the corrosive impact of seawater solution with a concentration of 24%, which in 1 dm^3^ contained 220 g of NaCl, 64 g of MgCl_2_, 15 g of KCl and 14 g of MgSO_4_. The composition of seawater was adopted based on literature data [8]. The mortars were kept in the seawater solution for a period of 90 days, whereas the accelerated carbonation process of the mortars was conducted in a carbonation chamber in 1% CO_2_ while keeping the air relative humidity equal to 60 ± 10% at a temperature of 21 ± 2 °C.

## 3. Testing Methods

The chemical composition of FBC and siliceous (CFA) fly ashes, as well as CEM I 42.5R cement, were determined based on the guidelines of PN-EN 196-2 standard “Methods of testing cement. Part 2: Chemical analysis of cement”. The compressive and flexural strengths of the cement–ash mortars were tested based on guidelines set forth in PN-EN 196-1:2016 standard. Tests of the aforementioned mortars were conducted on specimens with a standard size (40 × 40 × 160 mm). The consistency of the mortars was determined using a Novikov cone immersion test. A water–binder ratio of 5 ± 0.5 cm was selected in order to get the same cone immersion for all mortars.

The pozzolanic activity of FBC and siliceous fly ashes was tested using a standard method (in line with the guidelines of PN-EN 450-1:2012) and the Frattini test method [25]. The Frattini test method involved determination of ash activity derived from a ratio of the compressive strength of the mortars after their 7 day conditioning in water with a temperature of 21 °C, to the strength of mortars stored in water with a temperature of 21 °C for the first 3 days and then for the next 4 days in water with a temperature of 50 °C.

The compressive and flexural strength tests of the mortars were conducted for samples stored in tap water, seawater and the carbonation chamber in line with guidelines set forth in PN-EN 196-1:2016-07 standard, on specimens of size 40 × 40 × 160 mm after 90 days. After 24 h of curing, the samples were demoulded, and then, until compressive strength testing, they were stored in lab conditions at a temperature of 20 ± 2 °C for a period of 28 days. The mortars were then subjected to further testing to determine their durability.

The resistance of mortars to seawater and the carbonation process was determined based on linear changes using a Graff–Kaufman testing device, following the requirements of PN-B-19707:2013-10 [26]. The expanded uncertainty of measurement for linear changes equals: in water ±0.057%; sea water ±0.019%; carbonation ±0.017%. The expanded uncertainty of measurement for mass changes equals: in water ±0.6 g; sea water ±0.7 g; carbonation ±1.1 g.

X-ray tests of mortar samples were performed using a Philips X’PertSystem X-ray diffractometer. CuKα radiation was used. The measurement was carried out in the 2θ angle range from 5 to 60°.

The grain-size distribution of cements containing FCB and siliceous fly ashes was determined by means of a Mastersizer 3000 laser particle size analyser with a wet (liquid) dispersion method within a range from 0.01 to 1000 μm. Isopropyl alcohol was used as a dispersant.

The carbonation of mortars was tested using a phenolphthalein indicator method described in a draft of the concrete standard prEN 12390-12. Tests were performed on a fresh fracture surface of a specimen in a plane perpendicular with the surface of size 40 × 40 mm. Specimens were stored for 90 days in a climatic chamber in conditions with increased CO_2_ content (4%) and constant humidity (60%) at a temperature of 20 °C. After the curing period (i.e., after 7, 28, 56 and 90 days), specimens were subject to flexural strength tests, and then, by means of phenolphthalein solution, the carbonized surface area of the tested mortars was determined.

Mortar specimens subject to testing of calcium ion (Ca^2+^) leaching were prepared by their grinding in a mortar followed by their introduction into aqueous solutions of the acids HCl, H_2_SO_4_ and HNO_3_. Before titration, specimens were alkalized by adding 3 cm^3^ of KOH solution containing 120 g of KOH and 500 cm^3^ of distilled water, in the presence of 0.2 g of calconcarboxylic acid sodium salt. Solutions prepared this way were titrated with a solution of disodium versenate (C_10_H_14_N_2_O_8_Na_2_). Testing was conducted by keeping the same time intervals for each specimen, so that the time of a given mortar sample’s exposure to acid was 40 min [27].

Microscopic tests were conducted with the use of a field emission scanning electron microscope manufactured by Thermo Scientific, Quattro S model. Imaging of the microstructure was done in low-vacuum conditions with the application of an LVD detector (image display with secondary electrons). The analysis (non-model method) of EDS chemical composition was conducted through the application of a new generation detector by EDAX company.

## 4. Results and Discussion

### 4.1. X-ray Diffraction

In order to identify the structure and phases occurring in the FBC fly ashes (FBC I, FBC II and FBC III) and siliceous fly ashes (CFA) used for testing, X-ray diffraction tests were conducted. Table 2 presents the chemical composition and specific surface area, determined with the Blaine method, of fluidized bed combustion fly ashes (FBC) and siliceous fly ashes (CFA).

**Figure 1:**
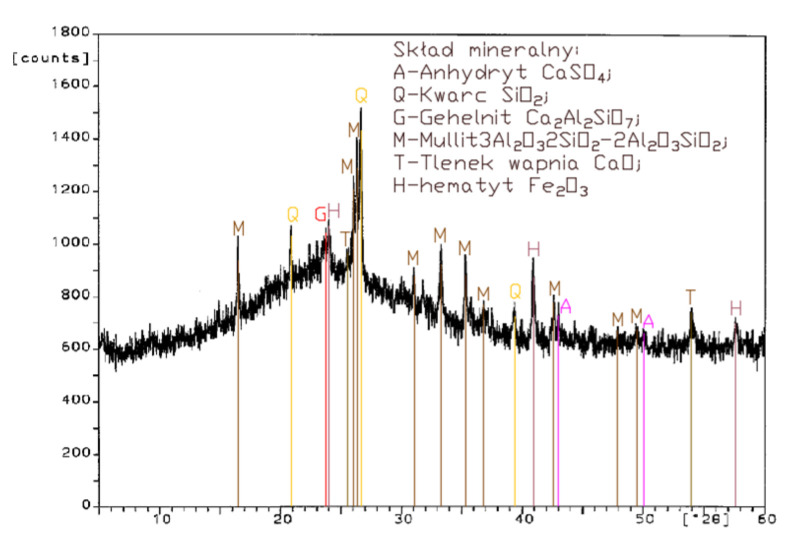
XRD pattern of conventional fly ash (CFA) from combustion of hard coal.

**Figure 2:**
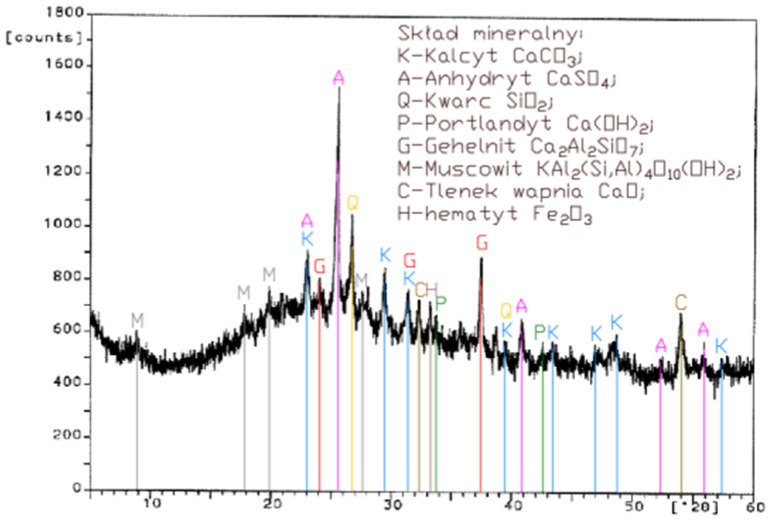
XRD pattern of fluidized bed combustion fly ash from combustion of lignite (FBC I).

**Figure 3:**
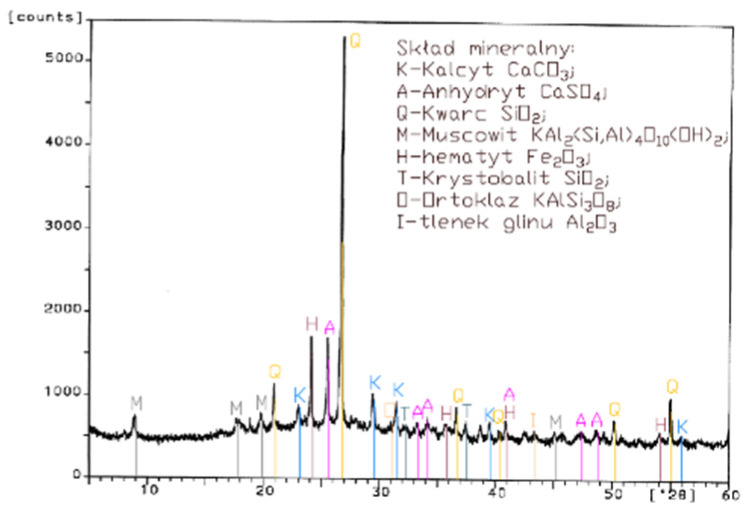
XRD pattern of fluidized bed combustion fly ash from combustion of hard coal (FBC II).

**Figure 4:**
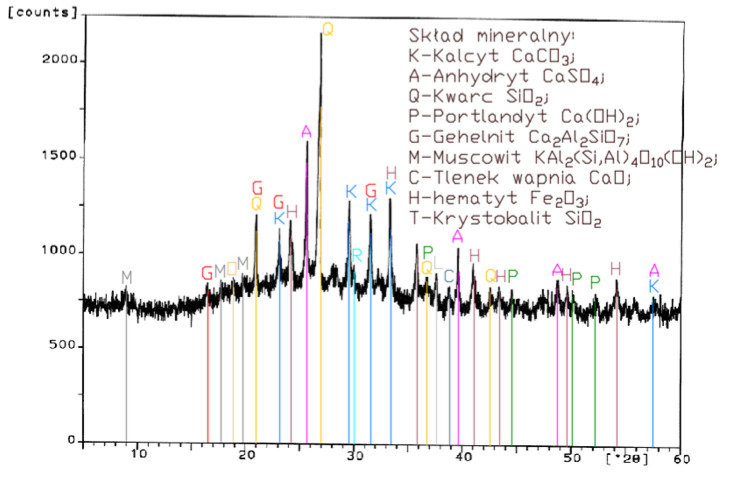
XRD pattern of fluidized bed combustion fly ash from combustion of hard coal (FBC III).

Analysis of the XRD test results (Figure 1, Figure 2, Figure 3 and Figure 4) and chemical composition (Table 2) of fly ashes that come from combustion in FBC and PF boilers demonstrated that FBC fly ashes had a disordered amorphous structure (Figure 2, Figure 3 and Figure 4).

A diffractogram of siliceous fly ashes (Figure 1) shows a high content of mullite phases, which is characteristic of ashes originating from PF boilers. On the other hand, no mullite is found in diffractograms of FBC fly ashes (Figure 2, Figure 3 and Figure 4), which is associated with the lower temperature of coal combustion in fluidized bed boilers (850 °C) compared to pulverized coal-fired boilers (1350 °C) [1,2,7,8].

Differences in the phase composition are characteristic and depend on the type of ashes used (FBC v siliceous fly ashes). Analysis of diffractograms of siliceous fly ashes (CFA) from hard coal combustion (Figure 1) indicated high quartz content, as well as the presence of hematite and calcium oxide. On the other hand, comparison of the phase composition of FBC I fly ashes from lignite combustion (Figure 2) with those of FBC II and FBC III fly ashes from hard coal combustion (Figure 3 and Figure 4) showed their different chemical and phase composition, depending on the type of coal fired.

FBC II fly ashes from hard coal combustion (Figure 3) showed the highest content of silica (SiO_2_) and increased quantities of hematite (Fe_2_O_3_), anhydrite (CaSO_4_) and calcite (CaCO_3_), as well as a large specific surface area according to Blaine method (8230 cm^2^/kg, Table 2). Meanwhile, FBC I fly ashes from lignite combustion (Figure 3), with the largest specific surface area out of all ashes tested, equal to 8750 cm^2^/kg (according to Blaine), had a high content of muscovite (KAl_2_(Si,Al)_4_O_10_(OH)_2_), anhydrite (CaSO_4_), quartz (SiO_2_) and gehlenite (Ca_2_Al_2_SiO_7_). In their case, the peak with the highest intensity was attributed to anhydrite. In the case of FBC III fly ash from hard coal combustion (with a specific surface area equal to 7870 cm^2^/kg), the peak with the highest intensity was attributed to quartz (Figure 4).

Tests of chemical composition (Table 2) also showed high content of quartz in FBC I from lignite combustion, which had not been explicitly found by means of X-ray testing (Figure 2). The high content of calcite in FBC fly ashes, compared to its content in siliceous fly ashes (CFA), results from the desulphurization method applied, i.e., using a sorbent in the form of ground limestone or dolomite. Anhydrite present in FBC fly ashes (FBC I, II, III, Figure 2, Figure 3 and Figure 4) is a product of the dehydration reaction of these products, which was indicated by [3,13,28].

Analyses of the chemical and phase composition of fly ashes showed that their properties may vary depending on the process by which they are acquired, including the type or quality of fuel used or the type of the combustion system (conventional or fluidized bed type), which is also pointed out in the literature [8,14,29,30,31].

### 4.2. Particle Size Distribution

**Figure 5:**
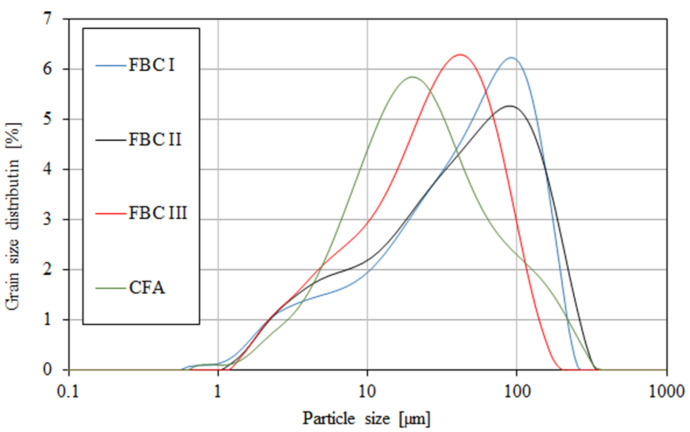
Particle size distribution of fluidized fly ash (FBC I, FBC II, FBC III) and conventional fly ash (CFA).

A grain-size distribution analysis by means of a particle size analyser (Table 3, Figure 5) showed that FBC and siliceous (CFA) fly ashes had different particle sizes in the range from 0 to ca. 400 μm (Figure 5).

**Figure 6:**
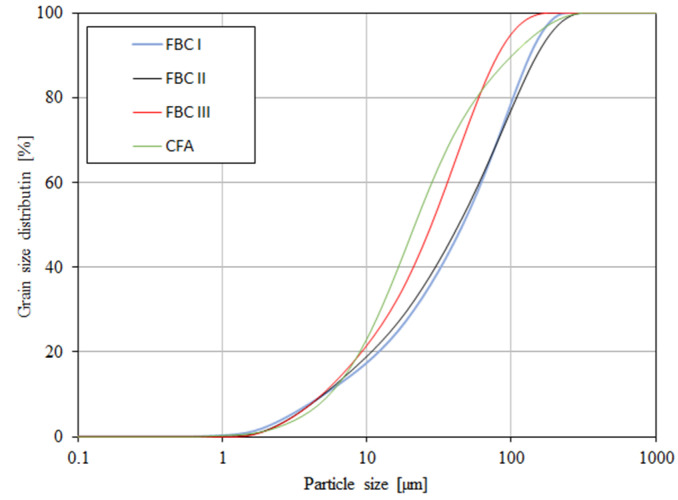
Accumulation curve of particle size distribution for FBC fly ash (FBC I, FBC II, FBC III) and fly ash from pulverized coal-fired boilers (CFA).

The content of larger particles (above 100 μm) showed a reverse relation, so the highest percentage content of particles >100 μm was shown for FBC I from lignite combustion, slightly lower for FBC II from hard coal, then lower for FBC III from hard coal and the lowest for CFA from combustion of hard coal (Table 3 and Figure 5). It was found that FBC I and FBC II fly ashes had a very similar particle size distribution for particles from 10 to 50 μm (Figure 5 and Figure 6).

### 4.3. Pozzolanic Activity According to “PN-EN 450-1”

**Figure 7:**
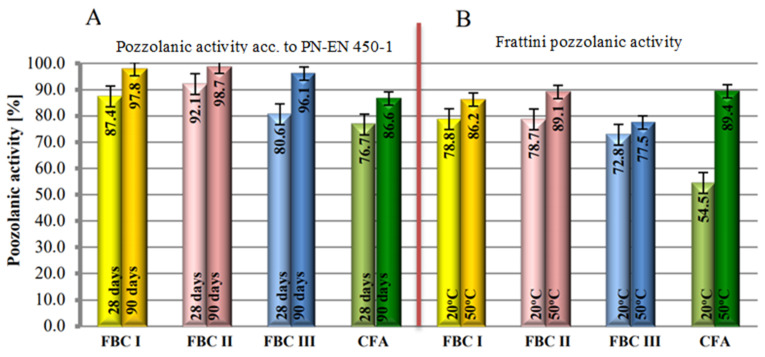
Activity of fluidized bed combustion fly ashes of different origins and conventional combustion fly ashes determined by the standard method according to PN-EN 450-1 (**A**) and the Frattini method (**B**).

The study involved determination of pozzolanic activity of fly ashes with the standard method [32] and Frattini method [25,33], the results of which are compared in Figure 7. Pozzolanic activity test results obtained with the Frattini method indicated that fluidized bed combustion fly ashes (FBC), cured in the normal conditions, i.e., 20 °C, showed higher pozzolanic activity than siliceous fly ashes (Figure 7).

This was determined by the presence of the amorphous phase and calcium oxide in FBC fly ash composition, which is also indicated in papers by Zheng [34], Brandt [8] and Rajczyk [4]. In addition, FBC I fly ashes from combustion of lignite, despite their pozzolanic properties, also showed hydraulic activity that was not demonstrated by siliceous fly ashes [35,36,37]. This was visible during the mortar setting reaction at the temperature of 20 °C, where the hydraulic nature of FBC fly ashes seemed to be dominant. The increase of the mortar curing temperature from 20 °C to 50 °C shows the intensification of the pozzolanic properties of siliceous fly ashes (CFA) from pulverized fuel-fired boilers, which in these conditions was confirmed by higher values of compressive strength for siliceous fly ash compared to FBC fly ash (Figure 7).

Differences in pozzolanic activity between FBC and siliceous fly ashes obtained herein may be explained by their phase composition, and in particular by the presence of dehydrated aluminosilicate substance as well as amorphous constituents. This was confirmed by diffractograms of FBC fly ashes, where a raised background was identified, indicating the presence of the aforementioned phases.

The presence of the amorphous phase in FBC fly ashes determines their properties, including their higher pozzolanic activity, which fact is also confirmed by [1,3,4,5,8]. Those authors also demonstrated the presence of calcium oxide and calcite, i.e., constituents that undergo hydration reactions, in the FBC fly ash composition.

Siliceous fly ash has high content of mullite (3Al_2_O_32_SiO_2_-2Al_2_O_3_SiO_2_) and quartz. This may explain the lower pozzolanic activity of siliceous fly ashes compared to the higher activity of FBC fly ashes in normal conditions (20 °C).

According to [35], the pozzolanic activity of fly ashes depends on the concentration of SiO_2_ and Al_2_O_3_ oxides they contain. Therefore, higher concentrations of silicon and aluminium oxides, demonstrated based on analysis of the chemical composition, indicate increased content of these oxides, which explains the higher reactivity of FBC fly ashes compared to the lower reactivity of siliceous fly ashes. Furthermore, FBC fly ashes, when reacting with portlandite (Ca(OH)_2_), form a larger quantity of the C-S-H phase in the form of gel and ettringite (AFt), which favours the increase of pozzolanic activity in cement mortars with FBC fly ashes [35,36].

Irrespective of the curing temperature (both at 20 °C and at 50 °C), the highest pozzolanic activity was demonstrated by FBC I fly ashes from lignite combustion, followed by slightly lower activity by FBC II fly ashes from hard coal combustion and the lowest activity by FBC III fly ashes from hard coal combustion.

The impact of the type and quantity of fly ashes of different origins on the durability of cement–ash mortars was determined further herein following the same procedure. Mortars, after their prior 28-day curing in water, were exposed to environmental aggressiveness factors, i.e., seawater and higher CO_2_ content in the process of accelerated carbonation, for a period of 90 days.

### 4.4. Consistency and Water Demand

**Figure 8:**
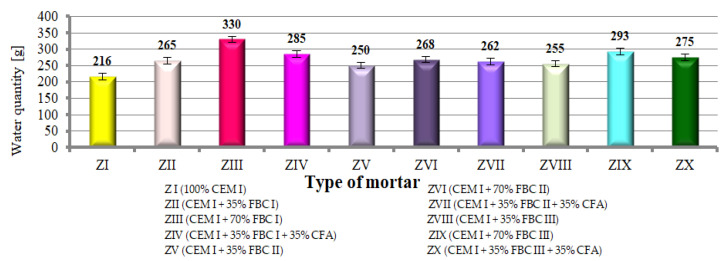
Quantity of water required to reach the consistency of the reference mortar for mortars containing FBC fly ashes and mixtures containing FBC fly ashes and CFA.

Testing the consistency of cement–ash mortars containing FBC fly ash of different origins, as well as mixes of FBC and siliceous fly ashes (from ZII to ZX, Figure 8) showed that mortars containing FBC fly ashes required an increased quantity of water to reach the consistency of the reference mortar (slump cone 5 ± 0.5 cm) compared to the reference mortar (ZI) made of Portland cement. The increased water demand for mortars with FBC fly ashes was also pointed out in [23,34,37,38,39,40,41].

It was demonstrated that mortars with the same quantity (35% by mass) of FBC I fly ash required more water (265 g H_2_O) to reach the consistency of the reference mortar compared to mortars with FBC III (255 g H_2_O). At the same time, cement with higher content of FBC fly ash (FBC I, II, III) (from 35 to 70% by mass) required a larger amount of water to reach the consistency of the reference mortar.

Results worth noting are those that indicate that by introduction of the mix composed of 35% by mass of FBC fly ash (I, II or III) and 35% by mass of siliceous ash (CFA) to cement, it was possible to reduce the water quantity by ca. 3 to 14% in mortars ZIV, ZVII and ZX, while keeping the same consistency (cone slump 5 ± 0.5 cm). When the same quantity (70% by mass) of FBC fly ash (FBC I or FBC II or FBC III) is used, but in the “homogeneous” form, in mortars ZIII, ZVI and ZIX, a larger quantity of water was required (which indicates the growth of water demand).

The above solution seems to be very essential due to the demonstrated possibility of a water–binder ratio reduction (Figure 8) in the case of mortars containing FBC and siliceous fly ashes, which would translate to improved performance parameters of mortars containing the ash mix, as demonstrated further herein.

It was shown that mortars containing 35 to 70% by mass of FBC fly ash demonstrated increased water demand, which grew along with the quantity of ash in the cement, and thus the content of fine particles up to 20 μm (Figure 5 and Figure 6, Table 3).

A different relation is observed for ZIV mortar containing 70% by mass of fly ash in the form of a mix. In this mortar, the presence of siliceous fly ashes (CFA), despite having a higher content by ca. 25% of fine particles below 50 μm (74.62 μm) in their composition compared to FBC I fly ash (49.6 μm), reduced mortar water demand by even 14%. In the case of ZIII mortar with 70% by mass of FBC I fly ash used in the homogeneous form, an increased demand for water was observed expressed by its water demand value. Differences observed were affected by the lower water demand of siliceous fly ashes caused by the structure of their grains, which have a spherical and regular shape enclosed in a glassy structure [6].

It should be emphasized that the issue of the increased water demand of cement–ash mortars is complex and depends on many factors, including the properties of the ashes themselves, such as their type, shape, particle size, specific surface area, content of carbon and free calcium oxide, and in the case of FBC fly ashes, their porosity [34,38,39,41].

### 4.5. Flexural and Compressive Strength of Cement–Ash Mortars

**Figure 9:**
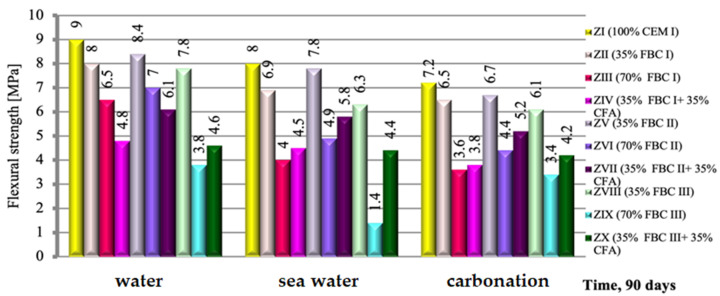
The flexural strength of mortars containing FBC fly ashes of different origins and mixtures of FBC and conventional fly ashes, cured in tap water, seawater and the carbonation process.

The flexural strength of a cement-based mortar (ZI) and cement–ash mortars (ZII-ZX), exposed to the impact of three different environments, i.e., tap water, seawater and accelerated carbonation under CO_2_ influence, were compared and presented in Figure 9. It was demonstrated that the exposure of cement–ash mortars (ZII-ZX) containing various quantities (from 35 to 70% by mass) and types to various aggressive environments for a period of 90 days resulted in lower (by ca. 23%) loss of flexural strength in seawater than in accelerated carbonation. On the other hand, cement–ash mortars exposed to the carbonation process showed a strength reduction of ca. 42% compared to the strength of those mortars in reference curing conditions (tap water, Figure 9).

It is characteristic that regardless of the type of the environment, the highest strength was achieved by mortars containing 35% by mass of FBC fly ashes, which due to the type of ashes used could be expressed by the following sequence: FBC II > FBC I > FBC III. This corresponds to the results of pozzolanic activity tests for those ashes (Figure 7).

An increase of FBC fly ash content in the cement to 70% by mass led to reduction of the mortars’ strength, regardless of the type of ash used and environmental conditions (tap water, seawater or CO_2_).

Furthermore, it has been demonstrated that through simultaneous introduction of the additive to cement in a quantity of 70% by mass, but in the form of a mix of FBC fly ashes (i.e.,: FBC I, FBC II and FBC III respectively) and 35% by mass of siliceous fly ash (CFA), a reverse effect can be obtained than in case of 70% by mass of FBC fly ashes in the “homogeneous” form, consisting in the increase of the flexural strength of the mortars. This is visible in particular for mortars ZIV, ZVII and ZX exposed to aggressive environments, both seawater and increased CO_2_. This was confirmed by the strength growth for mortars exposed to seawater, which amounted to 48.5% for ZX mortar, then 5.5% for ZIV mortar and 9.4% for ZVII mortar.

On the other hand, in the water environment, this phenomenon is observed only for FBC III fly ashes from hard coal, while mortars containing both FBC I fly ash from lignite and FBC II fly ash from hard coal used in the form of mixtures with siliceous fly ash, showed a reduction in strength (Figure 9).

In terms of fly ash origin, the highest strength, irrespective of the environment, was shown by mortars ZV, ZVI and ZVII, with FBC II fly ash from hard coal combustion; lower strength was shown by mortars ZII, ZIII and ZIV, with FBC I fly ash from lignite combustion; and the lowest was shown by mortars ZVIII, ZIX and ZX, with FBC III fly ash from hard coal combustion.

**Figure 10:**
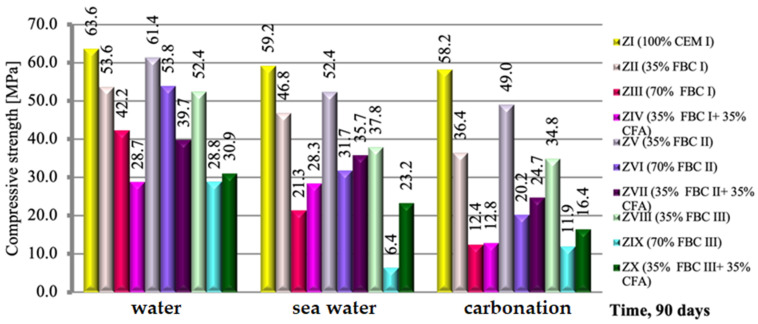
The compressive strength of mortars containing fluidized bed combustion fly ashes of different origins and mixtures of FBC and conventional fly ashes, cured in tap water, seawater and the carbonation process. (Expanded uncertainty of measurement for flexural strength equals: in water ±0.69; sea water ±0.87; carbonation ±1.0 (MPa)).

A relation analogical to flexural strength was observed in case of the compressive strength of those mortars, but with relatively higher strength values (Figure 10). Regardless of the environment of conditioning (tap water, seawater or CO_2_), after 90 days of testing, the compressive strength of mortars containing FBC I and FBC II fly ashes, from combustion of lignite and hard coal, respectively, was higher than that of mortars containing FBC III fly ash from hard coal combustion.

The highest compressive strength was achieved by mortars with 35% by mass of FBC II fly ash from hard coal combustion (Figure 10). The composition of those ashes (FBC II) showed the highest content of SiO_2_ (43.78%), SO_3_ and calcium oxide. Thus it was noticed that the higher the SiO_2_ content that was added to the cement together with FBC II fly ash from hard coal combustion, the higher compressive strength of the mortars exposed to the aggressive environment, i.e., sea water, was.

**Figure 11:**
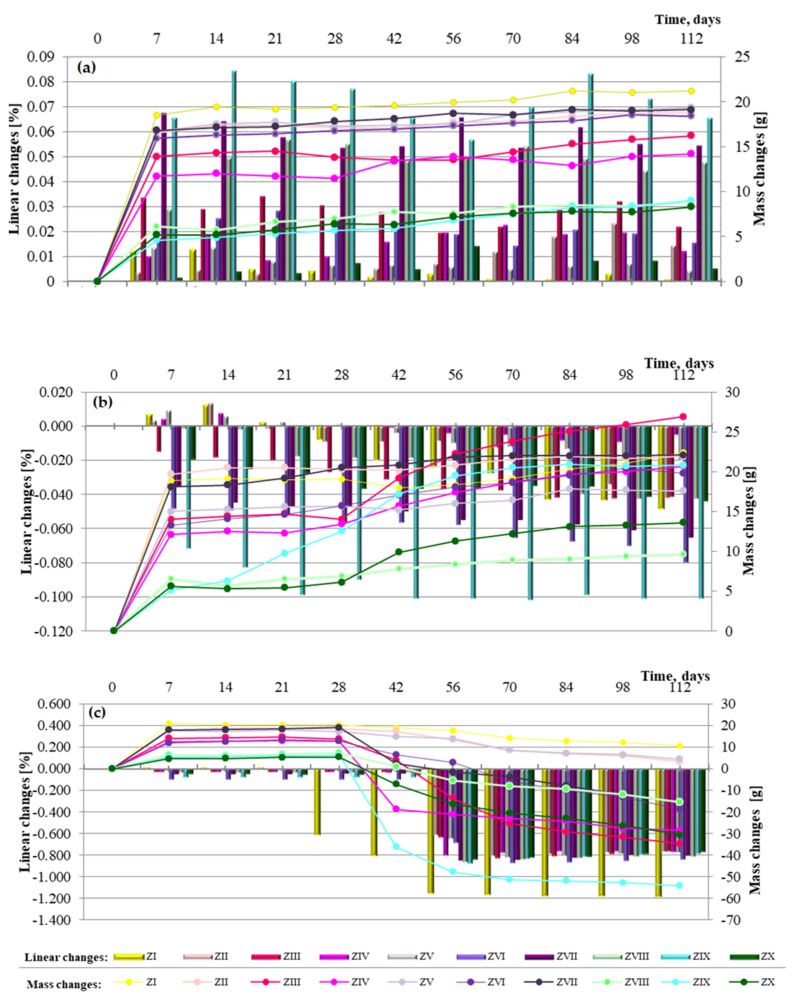
Linear and mass changes of cement mortars containing fluidized bed combustion fly ashes and mixtures of fly ashes from fluidized bed combustion and conventional combustion observed for a period of 112 days in the different environments: (**a**) in water, (**b**) in sea water and (**c**) in the carbonation.

**Figure 12:**
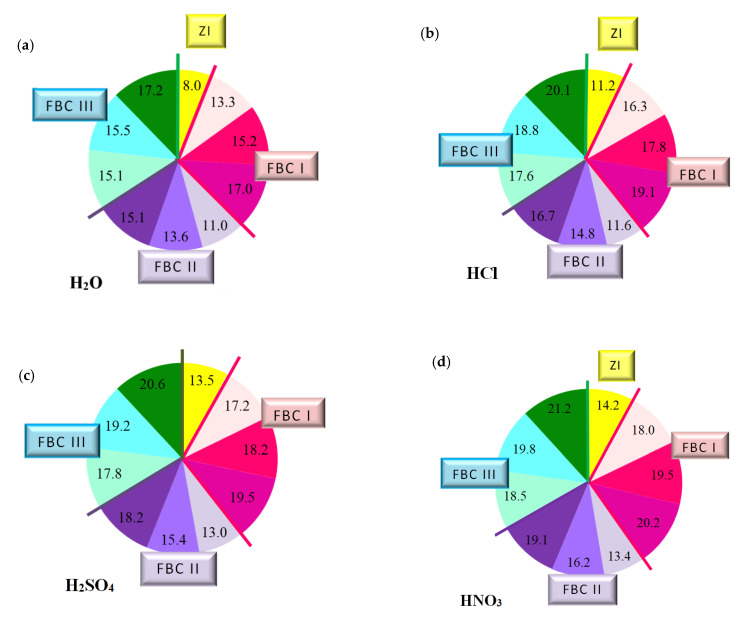
The quantity of leached calcium ions in mortars containing fluidized bed combustion fly ashes of various origins and mixtures composed of FBC fly ashes and conventional fly ashes exposed to the impact of the following environments: reference environment—distilled water H_2_O (**a**) and solutions of acids HCl (**b**), H_2_SO_4_ (**c**) and HNO_3_ (**d**), expressed in [mg/dm^3^].

**Figure 13:**
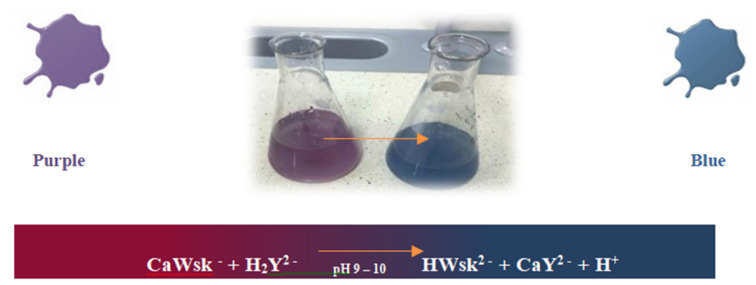
Transformations accompanying the leaching reaction of calcium ions (Ca^2+^) from cement–ash mortars in the environment of aggressive solutions of acids (HCl, H_2_SO_4_ and HNO_3_) under the influence of the disodium versenate indicator Na_2_H_14_C_10_O_8_N_2_∙H_2_O.

**Figure 14:**
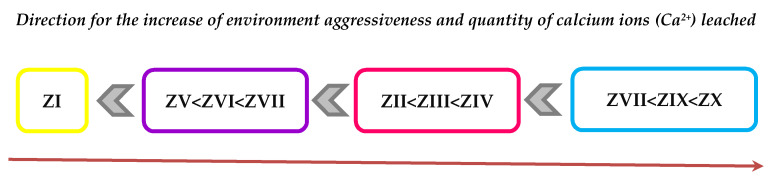
Relation between the quantity of calcium ions (Ca^2+^) leached and the type of ashes used in cement–ash mortars exposed to solutions of HCl, H_2_SO_4_ and HNO_3_.

**Figure 15:**
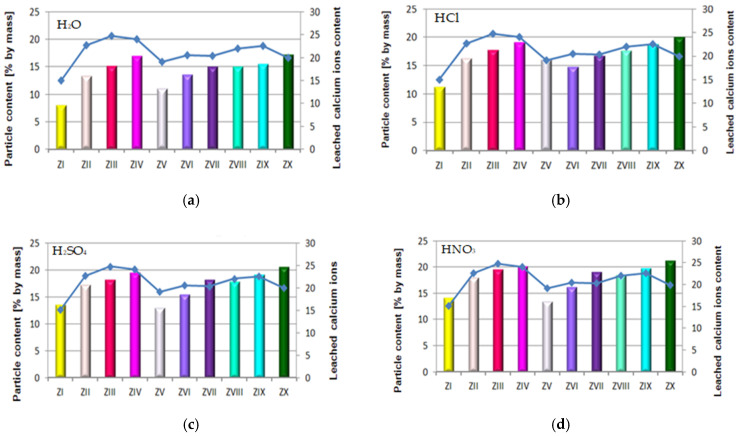
Relation between quantity of leached calcium ions (Ca^2+^) and content of particles >100 μm in mortars containing FBC fly ashes and CFA in different environments: (**a**) in H_2_O, (**b**) in HCl, (**c**) in H_2_SO_4_, (**d**) in HNO_3_.

**Figure 16:**
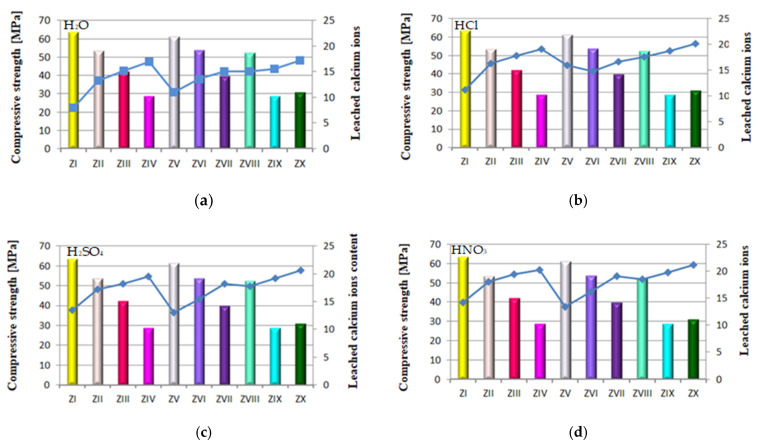
Relation between quantity of leached calcium ions and strength of mortars containing FBC fly ashes and CFA in in different environments: (**a**) in H_2_O, (**b**) in HCl, (**c**) in H_2_SO_4_ (**d**) in HNO_3_.

**Figure 17:**
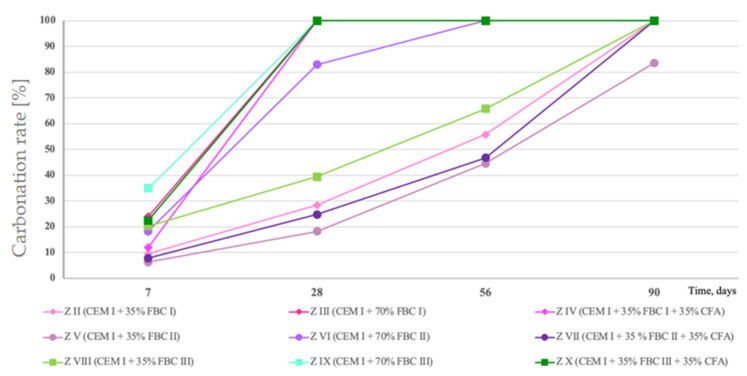
Percentage changes in carbonized surface area of mortars containing fluidized bed combustion fly ashes—FBC I, FBC II, FBC III.

On the other hand, the application of FBC III fly ash from hard coal to cement mortars resulted in significant deterioration of their performance parameters through reduction of strength (Figure 10) and resistance to aggressive environment (Figure 11, Figure 12, Figure 13, Figure 14, Figure 15, Figure 16 and Figure 17). This may be associated with the high values of unburned carbon particles and loss on ignition demonstrated for those ashes (Table 1) compared to other FBC fly ashes (FBC II and FBC I).

As in the case of the results of the flexural strength tests, it was also observed here that the use of the mixture composed of FBC and siliceous fly ashes (CFA) in mortars exposed to seawater and accelerated carbonation resulted in the growth of their compressive strength, in contrast to the tap water environment, where generally (except for the ZX mortar) a reduction in their strength was observed (Figure 10).

It should be emphasized that the flexural and compressive strength of mortars in CO_2_ atmosphere grew up to the 56th day. After 90 days in an atmosphere of 4% CO_2_, the strength of specimens tested decreased. Such behaviour may result from the increased amount of fine particles in FBC III fly ash, which additionally caused a high water demand for those mortars (ZVIII, ZIX and ZX). This may lead to a reduction in durability of mortars with FBC III in seawater and CO_2_. The destructive impact of carbon dioxide on concretes and mortars over a longer period was also demonstrated by other authors [8,12,13].

It was demonstrated that mortars with the FBC fly ash quantity increased to 70% by mass in the cement showed a drastic reduction of durability in aggressive environments, which caused a drop in their compressive strength by ca. 50% compared to mortars not exposed to seawater ions or increased quantities of CO_2_ during the accelerated carbonation process (Figure 10). In those conditions, in the case of the ZIX mortar with 70% of FBC III by mass, a reduction of compressive strength was observed by as much as 24% compared to the mortar with analogical composition cured in a tap water environment. The highest reduction of compressive strength was found in the case of mortars exposed to accelerated carbonation (in the carbonation chamber), including the reference mortar (ZI).

### 4.6. Determination of Linear and Mass Changes of Cement–Ash Mortars

Analysis of linear change test results for cement–ash mortars with FBC fly ashes, as well as mixes of FBC and siliceous fly ashes (Figure 11a–c), may indicate that seawater and accelerated carbonation have a destructive impact on mortars’ durability, but the scale and progress of the destructive impact of those corrosive environments depends on the composition of mortars.

Elongation of the linear dimensions of cement-based and cement–ash mortars being cured in the reference environment (i.e., tap water) with a simultaneous increase of their mass (Figure 11a) was characteristic.

In the case of mortars containing FBC fly ashes (ZII–ZX), elongation of their dimensions by ca. 0.01% was observed between the 56th and 84th days of specimen curing in tap water, which might indicate expansion caused by ettringite crystallization. In those conditions, the increased SO_3_ content in FBC fly ash may explain crystallization of the expansive ettringite, the presence of which, in the case of the ZIII mortar, was confirmed by the scanning microscopy testing.

In the aggressive environments (seawater and increased amounts of CO_2_ in the carbonation process), the opposite effect was observed, i.e., gradual reduction of mortars’ dimensions with a simultaneous gradual increase of their mass over time, up to 90 days (Figure 11b,c).

In the seawater environment, for the ZIX mortar containing 70% by mass of FBC III fly ashes, the greatest changes were observed in terms of its dimensions shortening, i.e., by ca. 0.1%, with simultaneously the greatest reduction of compressive strength (Figure 10).

The results of linear changes testing for mortars stored in the carbonation chamber show that changes observed until the 56th day of testing originated from drying. This is associated with the fact that until the 28th day, the mortars were cured in water and then exposed to CO_2_ in the carbonation chamber, where the air humidity was 60%.

The above is also confirmed by tests of mass changes showing a mass loss. It should be emphasized that linear changes of mortars kept in tap water and seawater did not exceed a value of 0.5%; therefore, tests were continued due to the larger size of specimens (40 × 40 × 160 mm), as well as the slower process of the seawater corrosion reaction than e.g., for the sulphate corrosion [14].

In general, in terms of the amount of ashes used, it can be stated that mortars containing 35% by mass of fluidized bed combustion fly ashes showed the least linear changes observed in terms of dimension elongation in the water environment, as well as the least tendency for dimension shortening in the seawater environment and the accelerated carbonation process. The largest changes of dimensions were obtained by mortars with the highest content of FBC fly ashes, i.e., 70% by mass. Introduction of an additional 35% by mass of siliceous fly ash to mortars containing 35% by mass of FBC fly ashes led to approximately a twofold reduction of linear changes observed towards shortening of their dimensions, which was demonstrated for each type of mortar containing ash mixtures (ZIV, ZVII and ZIX).

On the other hand, in terms of the type of ashes used, the least shortening of dimensions in aggressive environments was shown by mortars containing FBC II fly ashes from hard coal combustion, followed by FBC I from lignite combustion. Mortars containing FBC III fly ashes from hard coal in aggressive environments showed the greatest tendency for shortening of their dimensions.

Performed tests confirmed that the use of FBC and siliceous fly ashes definitely increased the mortars’ resistance to linear changes, both towards their elongation and their shortening. It was demonstrated that out of all mortars tested, the highest resistance to linear changes, both in the water environment and aggressive environments (seawater and carbonation process), was shown by the ZV mortar with 35% by mass of FBC II fly ashes from hard coal combustion.

### 4.7. Determination of Calcium Ions (Ca^2+^) Quantity Leached from Cement–Ash Mortars

The paper also includes tests performed to determine the quantity of calcium ions (Ca^2+^) leached from cement–ash mortars after 365 days of their storage in the water environment. For that purpose, the cement mortar (ZI) and cement–ash mortars (ZII–ZX) were exposed for 40 min to the direct impact of aggressive environments in the form of 1% solutions of the acids HCl, H_2_SO_4_ and HNO_3_, these being constituents of acid rain (Figure 12).

The transformation that accompanied the leaching reaction of calcium ions (Ca^2+^) from cement–ash mortars in the environment of aggressive solutions of acids (HCl, H_2_SO_4_, HNO_3_), a change of the solution colour from purple to blue in presence of the disodium versenate indicator Na_2_H_14_C_10_O_8_N_2_∙H_2_O, is presented in Figure 13.

It was demonstrated that the quantity of calcium ions leached depended on the mortar composition and the type of environment that mortars were exposed to. Determined quantities of calcium ions leached were the lowest for cement–ash mortars exposed to the reference environment—distilled water, then, higher values in the HCl solution, then in the H_2_SO_4_ solution, and the highest value in the HNO_3_ solution (Figure 12b–d). This may suggest that among the acid solutions used, the environment least destructive for cement–ash mortars is the hydrochloric acid solution, followed by the higher hazard demonstrated by the H_2_SO_4_ solution and the most aggressive hazard demonstrated by the solution of HNO_3_. This was confirmed because the greatest quantity of leached calcium ions (Ca^2+^) was obtained for cement–ash mortars exposed to nitric acid (HNO_3_).

In terms of the composition of mortars in each environment tested, in both the reference environment and solutions of acids, the smallest amount of calcium ions (Ca^2+^) leached was determined in ZI cement mortar, then a higher one for mortars ZV, ZVI and ZVII containing FBC II fly ash from hard coal, then the next higher value for mortars ZII, ZIII and ZIV containing FBC I fly ashes from lignite, and the highest value for mortars ZVIII, ZIX and ZX containing FBC III fly ashes from hard coal.

The above observations can be illustrated by a graph (Figure 14) indicating a relation between leached calcium ion quantity and the type of FBC fly ash used, regardless of the type of the aggressive environment, which is expressed in the following way:

It was demonstrated that depending on the type and quantity of FBC fly ashes, cement–ash materials with higher resistance to seawater and carbonation process could be obtained. The highest resistance to aggressive environment, and thus the most increased durability, was reached by mortars containing FBC II fly ashes from hard coal combustion, then a slightly lower value by mortars containing FBC I fly ashes from lignite combustion, and the lowest value by mortars containing siliceous and FBC III fly ashes from hard coal combustion. Alongside an increase of quantity (from 35% to 70% by mass) of FBC fly ashes in the cement, deterioration of the resulting mortars’ resistance to aggressive environments was observed. The highest resistance to aggressive environments was demonstrated by mortars with FBC II fly ash, and thus the lower content of calcium ions leached in these mortars indicated their low permeability, which was assured by hydration process products, such as C-S-H phase or ettringite (also confirmed with microscopic testing,). It was directly associated with the demonstrated high content of SiO_2_ and Al_2_O_3_ (Table 3) that showed increased reactivity [35].

As an example, the quantity of Ca^2+^ ions leached from the ZIX mortar containing 70% by mass of FBC III fly ashes from hard coal in the environment of solutions of acids was: HCl—18.8 mg/dm^3^, H_2_SO_4_—19.2 mg/dm^3^ and HNO_3_—19.8 mg/dm^3^, which indicated very low durability of that mortar in environment of acid rain. This was also confirmed by lower compressive strength values for that mortar (ZIX) obtained in the environments of seawater (6.4 MPa, Figure 10) and CO_2_ (11.9 MPa, Figure 10). On the other hand, the quantity of calcium ions leached from the ZIII mortar containing 70% by mass of FBC I fly ashes from lignite combustion was lower compared to that of the ZIX mortar; in solutions of acids, it was: HCl—17.8 mg/dm^3^, H_2_SO_4_—18.2 mg/dm^3^ and HNO_3_—19.5 mg/dm^3^. In that case, along with the increased resistance of that mortar (ZIII) to aggressive factors such as acid rains, higher values of strength parameters were observed (Figure 9 and Figure 10), as well as minor changes in linear and mass dimensions (Figure 11). In the case of the ZIV mortar containing the same amount of fly ashes, i.e., 70% by mass, but in that case FBC II fly ashes from hard coal, quantities of leached Ca^2+^ ions were the smallest (Figure 12). The above indicates that the ZXI mortar has the highest resistance to environment impact, which was also confirmed by its having the highest compressive strength values and the least linear and mass changes demonstrated compared to the ZIX and ZIII mortars.

The largest quantity of calcium ions (Ca^2+^) leached was obtained in the case of mortars containing mixtures of ashes composed of 35% by mass FBC fly ashes (FBCII/FBCI/FBCIII) and 35% by mass siliceous fly ashes, respectively, both in the reference environment and in 1% solutions of acids: HCl, H_2_SO_4_, HNO_3_—which might be in atmospheric precipitation in the form of acid rain (Figure 12). Out of these mortars, the lowest amount of Ca^2+^ ions was leached from the ZIV mortar containing FBC I fly ash, the next highest from ZVII with FBC II, and the highest from the ZX mortar containing FBC III fly ash.

The quantity of calcium ions leached from mortars exposed to 1% solutions of the acids HCl, H_2_SO_4_ and HNO_3_ may on one hand indicate the increased destructive impact of the aggressive environment leading to a larger amount of calcium ions (Ca^2+^) leached from hydration products of cement–ash mortars that grows along with the aggressiveness of the environment (HCl < H_2_SO_4_ < HNO_3_, Figure 12). On the other hand, however, the smaller quantity of calcium ions leached in the reference solution (H_2_O, Figure 12a) compared to the environment of hydrochloric acid (Figure 12b) corresponds with results from [3,5], indicating that expansion accompanying delayed ettringite formation in the presence of the increased content of SO_3_ from FBC fly ashes, may be delayed in the presence of chlorides originating from seawater. Such a situation affects washout of a higher quantity of calcium ions (Ca^2+^) in presence of Cl^-^ chlorides. The lack of expansive changes found in the dimensions of cement–ash mortars containing FBC fly ashes, demonstrated in tests of linear changes, may confirm the absence of conditions for delayed ettringite formation. Chloride ions cause a reduction in the durability of cementitious materials by changing their pH value to more acidic one, which causes the dissolution of hydrates and release of OH^-^ ions. First decalcification reactions occur in the C-S-H phase. The most durable constituent of the cement matrix is ettringite [12,30], which reacts last with the corrosive environment. Microscopic images taken confirmed the presence of ettringite in mortars exposed to seawater, without clear signs of expansion as demonstrated by ZIII mortars).

As demonstrated herein, in an environment of 1% HCl solution, the content of calcium ions (Ca^2+^) leached grew along with the increase of FBC fly ash content in the mortar based on CEM I 42.5 R Portland cement (Figure 12b), and it further grew in the presence of siliceous fly ashes. This analogical relation was also observed in aggressive solutions of the acids, i.e. sulphuric acid (H_2_SO_4_) and nitric acid (HNO_3_) (Figure 15c,d)

The above results corresponded to the observed relation indicating that alongside increased content of FBC fly ash in the cement, and thus the increased amount of fine particles of size <20 μm, the strength increases (Figure 16c,d).

The lowest content of particles up to 20 μm was shown by cements (CV, CVI, CVII) containing FBC II fly ash. In consequence, mortars of those cements showed the smallest quantity of calcium ions leached out of all cement–ash mortars tested, as presented in Figure 12a–d. However, when the content of particles >100 μm increased, the quantity of calcium ions (Ca^2+^) leached from cement–ash mortars grew (Figure 15a–d) and their strength decreased (Figure 16a–d). Analysis of obtained results (Figure 16a) indicated a direct relation between the quantity of leached calcium ions and the compressive strength of mortars, with FBC I and FBC III determined after 90 days of their curing in the water environment.

It was demonstrated that the higher the compressive strength of mortars stored for 90 days in water, the smaller the amount of calcium ions leached from those mortars was. In addition, as the FBC fly ash content (FBC I and FBC III) in mortars was increased from 35% to 70% by mass, a gradual reduction in their compressive strength was observed, as well as a larger quantity of calcium ions leached.

### 4.8. The Rate of the Carbonation Process by Means of the Phenolphthalein Indicator Test

Based on a test performed by means of a phenolphthalein indicator, the size of the fresh fractured surface area of mortars subject to the carbonation process was determined (Table 4, Figure 17).

Test results coincided with results of leached quantities of calcium ions, and they indicated that the fastest carbonation occurred in mortars with higher ash content—70% by mass of FBC fly ashes, i.e., in mortars ZIII, ZVI and ZIX, as well as in mortars composed of ash mixes containing 35% by mass of FBC fly ashes (FBC I or FBC II or FBC III) and 35% by mass of siliceous fly ash in mortars, i.e., in mortars ZIV, ZVII and ZX. The slowest process occurred in mortars containing 35% by mass of FBC fly ashes, i.e., ZII, ZV and ZVIII.

Mortars ZIII, containing 70% by mass of FBC I fly ashes from lignite combustion, and ZIX, containing FBC III fly ashes from hard coal combustion, showed the total carbonation of the specimen surface, which indicated a reduction of their pH value to a value below 8.2 after only 28 days of those mortars’ storage in the carbonation chamber (Figure 17 and Table 4). The highest resistance to carbonation in the CO_2_ environment out of mortars tested containing fly ashes was observed in case of the ZV mortar, containing 35% by mass of FBC II fly ash from hard coal combustion, with the highest SiO_2_ content (43.78%) and requiring the lowest amount of water to obtain the standard (reference) consistency. That mortar (ZV) also showed the highest resistance to the impact of aggressive environments, including acid rains. The quantity of calcium ions leached was in that case the lowest out of all mortars tested containing FBC fly ashes, and it was: in the presence of hydrochloric acid—11 mg/dm^3^, sulphuric acid—13 mg/dm^3^ and nitric acid—13.4 mg/dm^3^. The obtained smallest area affected by the carbonation progress on a cross-section of the mortar confirmed that the ZV mortar showed the highest resistance to aggressive environments, in both seawater and the carbonation process, as well as acid rains.

A slightly lower resistance to carbonation was demonstrated in turn by the ZVIII mortar containing 35% by mass of FBC III fly ashes from hard coal combustion. With that quantity of ashes, the ZII mortar containing FBC I fly ashes from lignite combustion showed to be the least resistant to carbonation.

It was demonstrated that application of the mixture composed of 35% by mass of FBC fly ashes and 35% by mass of siliceous fly ashes to mortars ZIV, ZVII and ZX was a favourable solution that hindered the carbonation progress compared to the effect obtained for mortars containing 70% by mass of “homogeneous” FBC (I, II and III) ashes. Increasing the amount of FBC fly ashes to 70% by mass in mortars ZIII and ZIX involved a higher risk of destruction, and thus lower resistance to aggressive environment, which was also indicated by a smaller cross-section area of the core of these mortars as ”unaffected” by corrosion (Figure 17 and Table 4).

### 4.9. Testing of Microstructure

**Figure 18:**
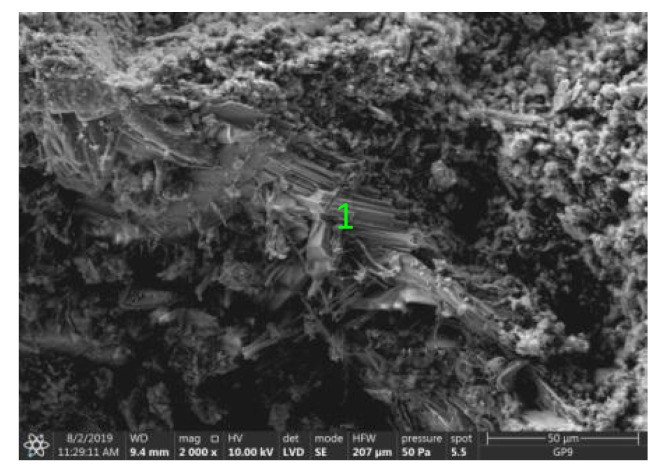
Microstructure of ZVIII mortar (FBC III) after 90 days of curing in seawater solution.

**Figure 19:**
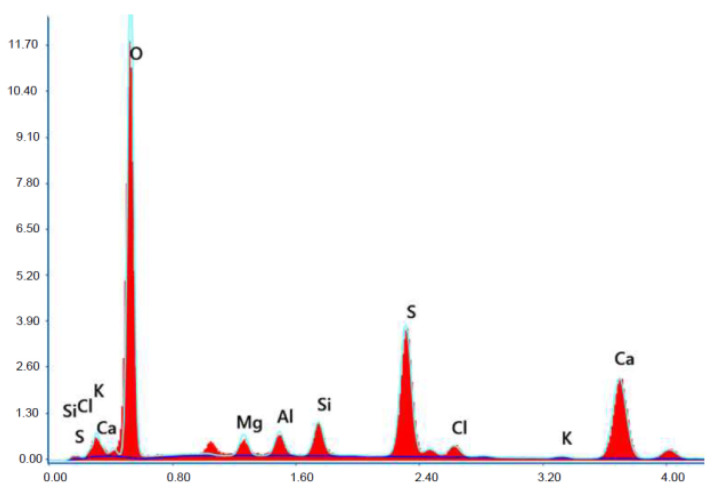
X-ray microanalysis (EDS) in point 1 from Figure 18.

**Figure 20:**
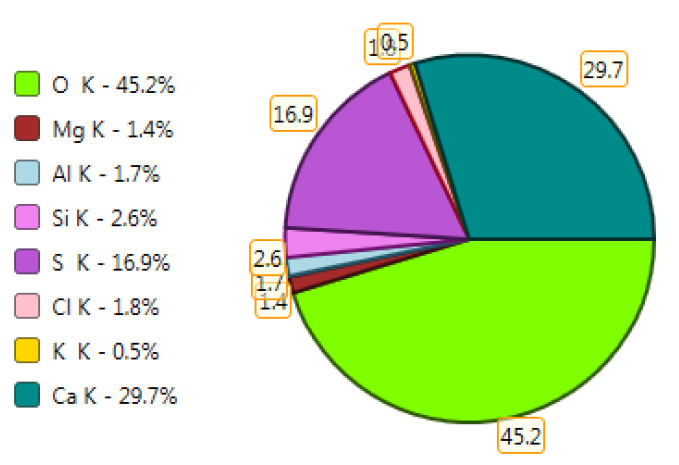
X-ray microanalysis (EDS) in point 1, percentage by mass of ingredients of the mineral.

**Figure 21:**
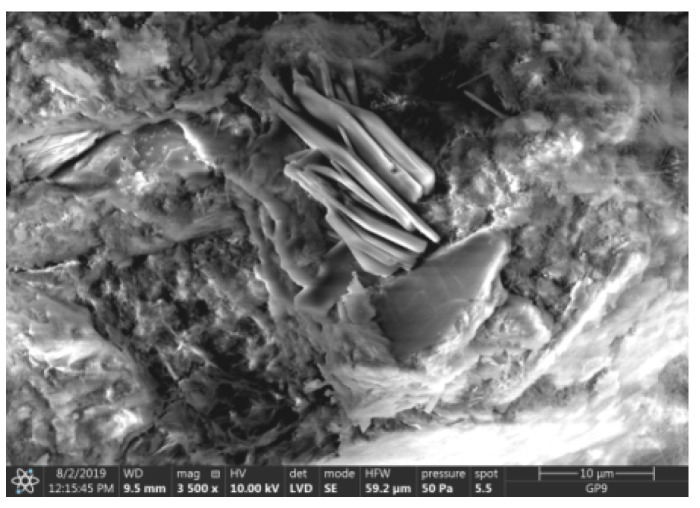
Microstructure of ZV mortar (FBC II) after 90 days of curing in seawater solution.

**Figure 22:**
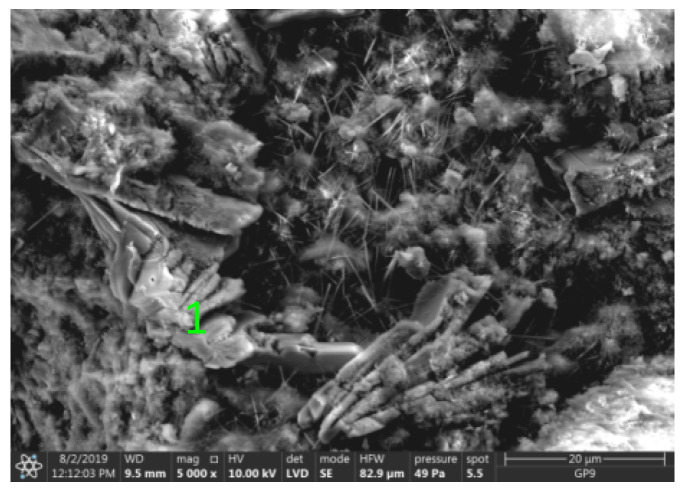
Microstructure of ZIX mortar (FBC III) after 90 days of curing in carbonation chamber.

**Figure 23:**
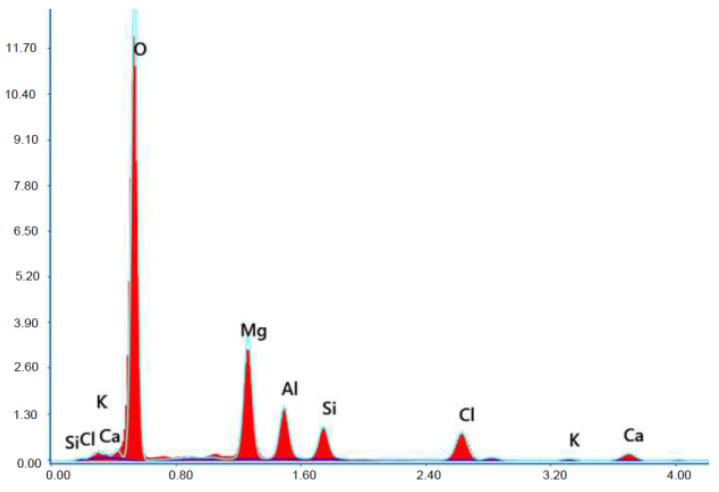
X-ray microanalysis (EDS) in point 1 from Figure 22.

**Figure 24:**
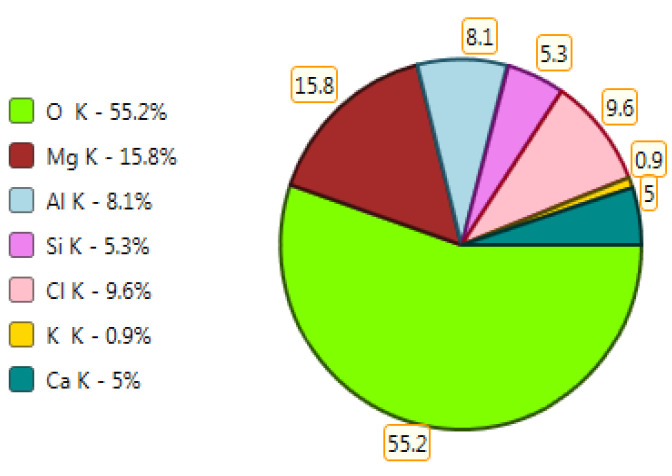
X-ray microanalysis (EDS) in point 1, percentage by mass of ingredients of the mineral.

**Figure 25:**
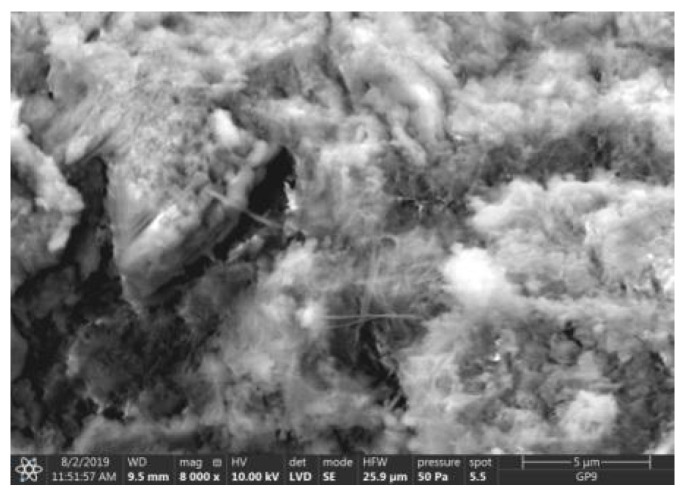
Microstructure of ZIII mortar (FBC I) after 90 days of curing in seawater solution.

Test results of cement–ash mortars’ microstructure conducted by means of scanning microscopy are shown in Figure 18, Figure 19, Figure 20, Figure 21, Figure 22, Figure 23, Figure 24 and Figure 25. It was demonstrated that in mortars tested, apart from typical products of cement hydration, there were also minerals present that are characteristic for the cement hydration reaction with FBC fly ashes.

The microscope image of the ZVIII mortar with FBC III fly ashes from hard coal (Figure 18) shows crystals of gypsum, the presence of which was confirmed by EDS analysis in the selected point 1 as shown in in Figure 19 and Figure 20. Gypsum minerals are rich in sulphur, the content of which in FBC III fly ashes was 4.07% by mass (Table 2). EDS microanalysis of gypsum in point 1 showed (Figure 19 and Figure 20) that the mineral contained 16.9% of sulphur (S) and 29.7% of calcium (Ca) by mass. On the other hand, Figure 21 shows the microstructure of the ZV mortar containing 35% by mass of FBC II fly ashes, where flat minerals of monosulphate (AFm) are visible, which according to [35] may be a product of the reaction of aluminates with ettringite. Figure 22 presents the microstructure of the ZIX mortar containing 70% by mass of FBC III fly ashes from hard coal combustion after the conditioning process in the carbonation chamber. Figure 22 shows the carbonized surface of the mortar in the vicinity of needle-like ettringite crystals and gypsum crystals, which are characteristic products of hydration of FBC fly ashes. However, in the figure showing the microstructure of ZIII mortars containing 70% by mass of FBC fly ashes (FBC I, Figure 25), unburned carbon particles and needle-like crystals of ettringite can be noticed.

Microscopic testing showed that mortars containing FBC fly ashes were rich in ettringite. Those mortars, in a later period of curing, showed increased linear changes towards elongation of dimensions and mass growth (Figure 11), which may confirm and indicate crystallization of the secondary ettringite.

Tests conducted showed that the higher amount of FBC fly ash in the cement significantly increased the water demand of cement–ash binders, thus creating a weakened cement–ash matrix, which, when exposed to seawater and the carbonation process, causes a reduction of mortars’ compressive strength. This was visible in particular in the case of mortars that contained 70% by mass of FBC fly ashes used in a ”homogeneous” form. Through replacement of 70% by mass of FBC fly ashes in the cement with a mixture composed of 35% by mass of siliceous fly ashes and 35% by mass of FBC fly ashes, reduction of the water demand was achieved by ca. 7%, which, as demonstrated herein, had a direct impact on the increase of durability and improvement of performance properties of those mortars even in aggressive environments.

The course of the carbonation process mainly depends on the water–binder ratio, and thus as claimed by [42] on the tightness of mortar microstructure, as well as the physical and chemical properties of FBC fly ashes. Therefore, the increase of FBC fly ash quantity above 35% by mass led to higher water demand of mortars, which also had consequences in their accelerated carbonation process and reduced resistance to aggressive environments (seawater and acid rain).

It was demonstrated that the mortar containing 35% by mass of FBC II fly ash from hard coal showed the highest resistance to the carbonation process and aggressive environments of seawater and 1% solutions of the acids HCl, H_2_SO_4_ or HNO_3_ (which are constituents of acid rain), which in this case was confirmed by the lowe quantity of calcium ions (Ca^2+^) leached. Additional hydration products of cement containing FBC fly ashes were responsible for the increased durability of cement–ash mortars, including C-S-H phase, ettringite, monosulphate and gypsum. This was also proved by the high compressive strength values of mortars containing FBC I, FBC II and FBC III ashes within a range from 77.5 MPa to 89.1 MPa, obtained with the curing temperature of those mortars increased to 50 °C, which was shown by the results of pozzolanic activity tests with the Frattini method (Figure 7).

Conformity of test results obtained with the results of [2,15] is also proved by the increased durability of mortars (ZV) containing FBC fly ashes, expressed in their higher strength and simultaneous minor linear and mass changes observed in the seawater environment. Simultaneously, the same mortars subject to the accelerated carbonation process showed reduced durability in an environment of higher CO_2_ content, which directly resulted in their lower strength and increased linear changes towards reduction of mortars’ dimensions and mass. The faster progress of carbonation was also confirmed by a phenolphthalein indicator test.

The destructive impact of corrosive environments on the durability of cement mortars is explained by the reaction of salt and carbon dioxide with constituents of the cement stone, as a result of which result of which calcium hydroxide (Ca(OH) is dissolved and easy soluble calcium salts (*Ca_n_R_2_*) are formed, the rinsing out of which weakens a mortar’s microstructure [13]. As a result, in a very general aspect, this leads to decalcification of the paste, which is associated with lower quantities of portlandite and decalcification of the C-S-H phase. As shown by test results herein, application of fly ashes from fluidized bed boilers (FBC II) from hard coal combustion as a constituent of mortars in the water environment may result in inhibition of calcium ions leaching from the cement matrix.

## 5. Conclusions

Based on test results obtained, it was demonstrated that:

- In normal curing conditions (20 °C) of cement–ash mortars, a hydraulic character originating from FBC fly ashes seemed to be dominant. However, increasing the environment temperature from 20 °C to 50 °C indicated a reverse relation determined by the pozzolanic character of siliceous fly ashes (CFA) from pulverized fuel-fired boilers, which in these conditions was indicated by the highest values of ash pozzolanic activity and growth in strength of mortars that contain these ashes.
- Application of FBC fly ashes in a quantity of 35% by mass led to obtaining the most favourable performance parameters of cement–ash mortars, assuring their high durability in the water environment, in aggressive precipitation and in conditions of accelerated carbonation. This was also confirmed by test results of mortars’ resistance to calcium ions Ca^2+^ leaching in an acid rain environment.
- Increasing content to 70% by mass of FBC fly ash in the homogeneous form in cement–ash mortars led to drastic deterioration of mortars’ performance properties and durability, including the quantity of calcium ions leached along with growing aggressiveness of the environment in the following sequence: HCl < H_2_SO_4_ < HNO_3_.
- Replacement of the binder containing 70% by mass of FBC fly ashes with a mixture composed of 35% by mass of siliceous fly ash and 35% by mass of FBC fly ash led to a significant reduction of the binder’s water demand and resulted in enhanced durability of cement–ash mortars, both in a neutral environment (H_2_O) and aggressive environments.
- The smallest quantity of leached calcium ions (Ca^2+^) was demonstrated by mortars with FBC II fly ash (from hard coal), showing the highest pozzolanic activity and specific surface area according to Blaine, i.e., 8230 cm^2^/g. The next higher quantity of calcium ions leached originated from mortars with FBC I from lignite combustion, and the highest from mortars containing FBC III from hard coal combustion.
- As fine particle content (up to 20 μm) in the cement originating from FBC fly ashes increased, leaching of calcium ions (Ca^2+^) from cement–ash mortars decreased and their strength grew. A reverse relation was observed for increased content of particles larger than100 μm.
- The most favourable performance parameters and increased durability in aggressive environments (acid rain and seawater) and the carbonation process were observed for mortars containing FBC II fly ashes from hard coal, which was reflected in: the highest values of flexural and compressive strength, the lowest linear and mass changes, the smallest amount of calcium ions (Ca^2+^) leached (in the environment of H_2_O and in 1% solutions of the acids HCl, H_2_SO_4_ and HNO_3_), and the smallest carbonized surface area of the mortar. Slightly worse parameters were observed in the case of mortars containing FBC I fly ashes from lignite combustion. In the presence of FBC III fly ashes from hard coal combustion, deterioration of mortars’ performance parameters was the greatest.
- The increased quantity of calcium ions (Ca^2+^) leached from cement–ash mortars in 1% solution of HCl, compared to the smaller quantity leached from mortars exposed to a water environment, remained in a close relationship with the observed lower expansion of these mortars in seawater than in tap water based on tests of linear changes.
- In the future, FBC fly ashes containing a higher amount of SO_3_ may become a main ingredient that guarantees delayed deterioration of coastal structure elements over time.

## Figures and Tables

**Table 1:** Composition of mortars with fluidized bed combustion fly ash (FBC I, FBC II, FBC III) and siliceous fly ash (CFA).

Type of Mortar	CEM I 42.5 R (g)	FBC * (g)	CFA * (g)	Aggregate (Sand, Fraction 0–2 mm) (g)	Water (g)
**Z I** (100% CEM I 42,5R)	450	-	-	1350	216
**ZII** (CEM I + 35% FBC I)	292.5	157.5	-		265
**ZIII** (CEM I + 70% FBC I)	135	315	-		330
**ZIV** (CEM I + 35% FBC I + 35% CFA)	135	157.5	157.5		285
**ZV** (CEM I + 35% FBC II)	292.5	157.5	-		250
**ZVI** (CEM I + 70% FBC II)	135	315	-		268
**ZVII** (CEM I + 35% FBC II + 35% CFA)	135	157.5	157.5		262
**ZVIII** (CEM I + 35% FBC III)	292.5	157.5	-		255
**ZIX** (CEM I + 70% FBC III)	135	315	-		293
**ZX** (CEM I + 35% FBC III + 35% CFA)	135	157.5	157.5		275

* FBC—fluidized Fly ash * CFA—conventional Fly ash.

**Table 2:** Chemical composition of fluidized bed combustion fly ashes (FBC I, FBC II, FBC III) and siliceous fly ashes (CFA).

Ingredient	FBC I	FBC II	FBC III	CFA	CEM I 42.5R
LOI	2.39	4.03	5.71	1.81	2.57
SiO_2_	39.37	43.78	35.87	40.20	19.79
Fe_2_O_3_	3.89	4.57	9.73	2.00	2.98
Al_2_O_3_	32.77	21.09	22.69	6.00	5.76
CaO	10.67	15.92	16.01	43.20	62.28
MgO	1.44	1.61	1.88	4.70	1.71
SO_3_	3.09	4.68	4.07	0.10	2.62
Na_2_O_eq_	1.22	0.75	1.02	-	0.75
Specific surface area acc. to Blaine [cm^2^/g]	8750	8230	7870	3800	4000
***FBC I**—fluidized bed combustion fly ash from lignite combustion in power plant I* ***FBC II**—fluidized bed combustion fly ash from hard coal combustion in power plant II* ***FBC III**—fluidized bed combustion fly ash from hard coal combustion in CHP plant III* ***CFA**—conventional fly ash from power plant III* ***CEM I 42.5R**—Portland cement CEM I 42.5R*					

**Table 3:** Particle size distribution of fluidized fly ash (FBC I, FBC II, FBC III) and conventional fly ash (CFA).

Particle Size Distribution (%)				
	FBC I	FBC II	FBC III	CFA
**D (<1 μm)**	0.31	0.00	0.00	0.27
**D (<10 μm)**	17.28	18.75	21.24	22.63
**D (<50 μm)**	49.63	51.50	69.49	74.62
**D (<100 μm)**	78.01	76.48	94.62	89.46
**D (>100 μm)** **from 100 to 480 μm**	22.00	23.52	5.38	10.54

**Table 4:** Surfaces of mortars containing FBC I, FBC II and FBC III fly ashes, after testing the carbonized surface with the phenolphthalein solution.

Type of Mortar	After 7 Days	After 28 Days	After 56 Days	After 90 Days
**ZII** (CEM I + 35% FBC I)	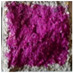 9.5%	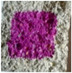 28.4%	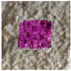 55.8%	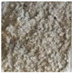 100%
**ZIII** (CEM I + 70% FBC I)	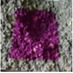 24%	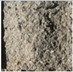 100%	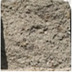 100%	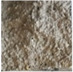 100%
**ZIV** (CEM I + 35% FBC I + 35% CFA)	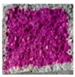 12.0%	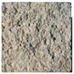 100%	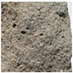 100%	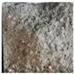 100%
**ZV** (CEM I + 35% FBC II)	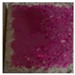 6.3%	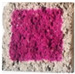 18.2%	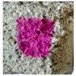 44.6%	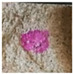 83.6%
**ZVI** (CEM I + 70% FBC II)	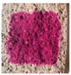 18.3%	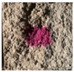 83.0%	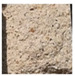 100%	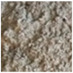 100%
**ZVII** (CEM I + 35% FBC II + 35% CFA)	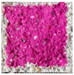 7.8%	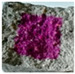 24.8%	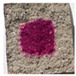 46.8%	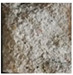 100%
**ZVIII** (CEM I + 35% FBC III)	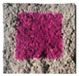 20.3%	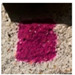 39.4%	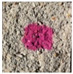 65.8%	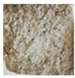 100%
**ZIX** (CEM I + 70% FBC III)	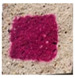 35.0%	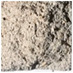 100%	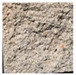 100%	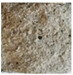 100%
**ZX** (CEM I + 35% FBC III + 35% CFA)	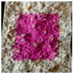 22.5%	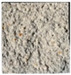 100%	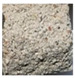 100%	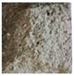 100%

## Data Availability

Data is contained within the article.
